# Managing Livestock Species under Climate Change in Australia

**DOI:** 10.3390/ani1040343

**Published:** 2011-10-19

**Authors:** S. Niggol Seo, Bruce McCarl

**Affiliations:** 1 Faculty of Agriculture, Food, and Natural Resources, The University of Sydney, Sydney, NSW 2006, Australia; 2 Department of Agricultural Economics, Texas A&M University, College Station, TX 77843, USA; E-Mail: mccarl@tamu.edu

**Keywords:** climate change, adaptation, livestock species, Australia

## Abstract

**Simple Summary:**

World communities are concerned about the impacts of a hotter and drier climate on future agriculture. By examining Australian regional livestock data on sheep, beef cattle, dairy cattle, and pigs, the authors find that livestock production will expand under such conditions. Livestock revenue per farm is expected to increase by more than 47% by 2060 under the UKMO, the GISS, and a high degree of warming CSIRO scenario. The existence of a threshold temperature for these species is not evident.

**Abstract:**

This paper examines the vulnerabilities of major livestock species raised in Australia to climate change using the regional livestock profile of Australia of around 1,400 regions. The number of each species owned, the number of each species sold, and the aggregate livestock revenue across all species are examined. The four major species analyzed are sheep, beef cattle, dairy cattle, and pigs. The analysis also includes livestock products such as wool and milk. These livestock production statistics are regressed against climate, geophysical, market and household characteristics. In contrast to crop studies, the analysis finds that livestock species are resilient to a hotter and more arid climate. Under the CSIRO climate scenario in which temperature increases by 3.4 °C, livestock revenue per farm increases significantly while the number of each species owned increases by large percentages except for dairy cattle. The precipitation reduction by about 8% in 2060 also increases the numbers of livestock species per farm household. Under both UKMO and GISS scenarios, livestock revenue is expected to increase by around 47% while the livestock population increases by large percentage. Livestock management may play a key role in adapting to a hot and arid climate in Australia. However, critical values of the climatic variables for the species analyzed in this paper are not obvious from the regional data.

## 1. Introduction

The earth has been warming, according to the Intergovernmental Panel on Climate Change (IPCC), due in large part to anthropogenic activities such as fossil fuel uses, agricultural emissions and land use changes [1]. The impacts of climate change are expected to vary geographically with the largest impacts felt in low latitude developing countries where agriculture plays a key economic role [2-5]. Climate change is likely inevitable and thus adaptation is becoming a widely discussed policy option [6]. Recently, economic research has started to examine possible adaptation options [7-12] and this area of work will continue to grow given the inevitable need for adaptation to climate change [6,13-15].

Empirical studies indicate that livestock management may be quite resilient against global warming and arid conditions due to climate change. For example, Seo and Mendelsohn found African farmers adapted by choosing goats and sheep more frequently when climate becomes hotter [7]. In Latin America, findings indicate farmers rely on sheep more than beef cattle under hotter climates [8]. Also, integrated crop and livestock management is found to be more common under hotter conditions than farms specializing in only crops [9,12] with integrated farms predicted to suffer only half the damage in land values from the 21st century climate change [10]. In the U.S., findings are that hotter conditions cause animal breeds to be adapted toward more of a Brahman influence [16] when stocking rates fall and land shifts from cropland to pasture [17]. This economically based literature has an animal science parallel in which the climate drivers leading to changes in animal breed selection are debated [18]. Animal scientists have also long investigated relationships between climate factors and animal production [19-21]. Finally, animal science and more general climate literature indicate that adaptive capacity of different livestock species to climate stress varies and that management options are available to facilitate adaptation [22,23]. Thus, in heavy livestock producing countries, climate change adaptation may be needed and alternatives are available. Policy makers, farmers and others may wish to carefully examine livestock sensitivity and adaptation in low-latitude countries which are both heavily dependent on livestock management and argued to be highly vulnerable to climate change [24-26].

Australia is a country that relies heavily on livestock for domestic consumption and exports. In such a setting, a closer examination of the interplay between climate, climate change and livestock is an important issue but one that is often left out of policy discussions [27]. The mix of livestock species and breeds that would better adapt to hotter conditions due to genetic components or other reasons is one of the fundamental adaptation research questions for scientists [18]. Research on livestock adaptation would provide important information paralleling findings on the crop side where crops are reported to be differentially vulnerable to temperature increases and rainfall reduction [2,28-30].

This paper examines the ways livestock management in Australia varies with climate as an input to identifying adaptation strategies and their effectiveness. Regional livestock data are obtained from the Australian Bureau of Statistics (ABS) [31], the Australian Bureau of Agricultural and Resource Economics (ABARE) [32] with climate data drawn from the Climate Research Unit [1,33]. Soils data are from Zobler's World File for Global Climate Modeling [34,35] and vegetation and land cover information is from the Matthews' Vegetation and Land Use Data [36].

These data are examined to see climate influences on livestock populations and revenues from sales of sheep, beef cattle, dairy cattle, and pigs. Regressions were run against climatic variables, along with geophysical (soil and geographic), market and household characteristic factors. The availability of feed from crop production is also examined. Marginal impacts of temperature and precipitation are examined by livestock species. In turn the effects of projected 2060 climate scenarios from the Australian Commonwealth Scientific and Industrial Research Organization (CSIRO) [1,37] and the IPCC A2 scenarios from the GISS (Goddard Institute for Spatial Studies) [38] and UKMO (United Kingdom Meteorology Office) [39] models. Specifically, the GISS-ER model and HadGEM1 model of the UKMO documented in the IPCC Data Distribution Center are examined.

This paper proceeds as follows. A brief description of the theory and methodology used to examine the variation of livestock revenue and species across climate zones is provided in the next section. The following section presents a detailed description of the data set. In the subsequent sections, empirical estimation results and the consequences of the projected 2060 climate scenarios are analyzed. The paper ends with discussions and conclusion.

## 2. Methods

To examine the effects of climate on livestock species choice and revenue, a statistical estimation approach is used with climate and non-climate signals to which a farmer responds controlled as explanatory variables. Classical economic profit maximizing is assumed. Suppose a farmer chooses a livestock species mix from a set of available animal species to maximize profit considering the given climate, geophysical, market and household factors. Then, the choices of species across the landscape would capture the best outcomes as climate and other conditions are varied [40].

More formally, let *π_j_* be the profit from species *j*. Then, a livestock producer's problem can be written as follows:

(1) $$\text{Max}\sum_{j}\pi_{j}(E,G,M,H)$$

where *E* denotes climate variables, *G* the geophysical conditions (soils and geography), *M* economic market conditions, and *H* household characteristics.

The maximization determines the choices of species and the optimal mix of inputs and outputs. The maximized profit can then be written as a function of exogenous variables as follows:

(2) π*=π(E,G,M,H)

The maximum profit from each species can be estimated as follows [41]:

(3) $$\pi_{j}=\pi (E,G,M,H|D_{j}=1)$$

where *D_j_* is a dummy variable that indicates the choice of species *j*.

Among the factors that determine profit for livestock species *j*, climate (*E*) is the primary concern of this analysis; *G* represents a great diversity of geophysical conditions involving soils and geography across Australia; *M* captures economic market conditions such as access to livestock export ports as well as urban markets; H reflects household characteristics such as whether the farm has complementary feed from grain productions or number of vehicles owned.

The impact of a change in climate on species *j* can be measured as the difference in the estimated profits under two climate conditions. That is, if climate is changed from *E*_1_ to *E*_2_, then the impact is measured as follows:

(4) $$\Delta \pi_{j}=\pi_{j}(E_{2})-\pi_{j}(E_{1})$$

Limitations due to the available data sets as well as detailed specifications are discussed in the ensuing sections.

## 3. Description of Data

Livestock ownership and revenue data were drawn from the 2006 regional profile of Australia compiled by the Australian Bureau of Statistics (ABS) for around 1,400 local areas [31]. These data contain the reported numbers of beef cattle, dairy cattle, sheep/lambs, and pigs at each locality, and also contain data on revenue from livestock slaughter and livestock products such as wool and milk.

Livestock sale data came from the Australian Bureau of Agricultural and Resource Economics [32]. That Bureau reported data for the years 1990 to 2009 for 34 sub-regions in terms of average statistics based on surveys conducted using random sampling. The data set also reported livestock product volume and included information on socio-economic variables such as age of the manager and off-farm labor hours.

Climate data in terms of monthly average temperature and precipitation were drawn from the Climate Research Unit. These data are at the scale of 10 arc minute, based on more than 27,000 global weather stations [33]. In the Southern Hemisphere, data were aggregated into summer (December, January, and February) and winter (June, July, and August) observations. Using the ArcView toolbox, the centroid of each locality in the ABS data was calculated and the climate data were interpolated to that centroid using a shortest distance method.

Soil data were from the Zobler's World File for Global Climate Modeling [34]. The data set reports a dominant soil for each grid cell of the globe. Most frequently found Australian soils are Acrisols, Cambisols, Ferralsols, Phaeozems, Lithosols, Luvisols, Nitosols, Podzols, Arenosols, Regosols, Solonetz, Andosols, Vertisols, Planosols, Xerosols, Yermosols, and Solonchaks. Many local areas are also near a water body such as the Murray Darling Basin or along the coasts.

Socio-economic variables were obtained from the ABS regional profile of Australia [31] consisting of information on human population density and distribution as well as household characteristics such as vehicle ownership, racial composition, female ratio, and building construction at the local level.

**Figure 1:**
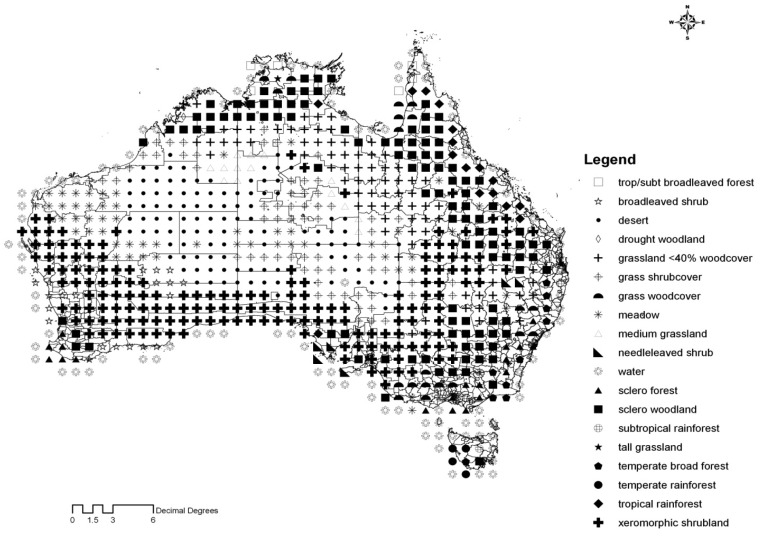
Major land covers in Australia.

Finally, major vegetation and land cover data were drawn from the Global Land Use and Vegetation data set [36]. As shown in Figure 1, Australia contains diverse ecological systems and land covers [42]. Along the eastern coast of Australia are mostly temperate broadleaved forests. Adjacent to the forests are sclerophyllous woodlands. Further inlands are xeromorphic shrublands. Central parts of Australia are occupied by grasslands and deserts. Southern Australia is mostly made up of xeromorphic shrublands. Western Australia is a mixture of forests, woodlands, and shrublands in the south, and a combination of shrublands and meadow in the north. Northern parts are drought affected deciduous woodlands, sclerophyllous forests, and grasslands.

## 4. Empirical Results

State and Territory level descriptive statistics computed across the local areas with reported livestock populations are summarized in Table 1. Summer temperatures range from 15 °C in Tasmania (TAS) to 28 °C in the Northern Territory (NT). Winter temperature is as low as 5 °C in the Australian Capital Territory (ACT). The driest State is Western Australia (WA) in the summer and NT in the winter. Xerosols are dominant soils in South Australia (SA) while Planosols are most common in WA and NT. On average, ACT is located in higher altitudes. The proportion of the population that are females is lower than 0.5, especially in NT and ACT. The number of passenger vehicles per 1,000 people is larger in New South Wales (NSW) and Victoria (VIC). New building approvals are larger in VIC and WA.

Average livestock sales per farm during the 1990 to 2009 period summarized by species/product and sub-region are discussed in the next section [43]. Averaged over the 20 year period, beef cattle sales are largest in the Barkly Tablelands in the NT with 3,273 cattle per year per farm. The Kimberly region of WA sells around 1,700 cattle per year. The smallest sale is recorded at the wheat belt in WA with 7 per farm. Sheep sale is largest in the Far West in NSW with 1,284 head per farm and in Central and South Wheat Belt in WA with 1,339 head per farm. Total sale of wool is well matched with the sale of sheep, but is second largest in North Pastoral region of South Australia (SA). There is limited sale of sheep or wool in the regions of NT and WA where cattle sales are greater.

To examine how climate influences the variation in beef cattle, dairy cattle, sheep, and pig populations, the number of animals by species is regressed against seasonal climatic variables, physical, socio-economic factors and regional household characteristics with the results presented in Table 2, Table 3, Table 4, and Table 5. For each species, we run two regressions with and without household characteristic variables. The dependent variable is the log of the number of animals by species per 1,000 rural people in the area. Results for the sheep regression are presented in Table 2. Focusing on the full model, summer temperature and precipitation turn out to be highly significant with P-values less than 0.01. The model R-sq is high at 0.72. The positive quadratic term of summer temperature indicates a U-shaped response. That is, when summer temperature rises beyond a certain point, the number of sheep owned increases.

The results indicate that soils and geographic variables are relevant factors in raising sheep. The sheep number owned is higher in an area which is farther from the coast. The number increases at higher altitudes. Fewer sheep are raised on Acrisols, Cambisols, and Xerosols soils while more sheep are raised in Lithosols and Vertisols. Socio-economic variables are also significant. When the female ratio in the human population rises, there are fewer sheep raised. The number of passenger vehicles and building approvals is negatively associated with sheep ownership. But, when there are more pension receivers, sheep ownership is larger.

The regression results for beef cattle are presented in Table 3. Again, summer temperature turns out to be critical with a positive quadratic term indicating a U-shaped response. The number of beef cattle increases in larger towns and at high elevations. Fewer beef cattle are raised on Xerosols whereas more beef cattle are raised on Ferralsols, Podzols, and Vertisols. Fewer beef cattle are raised in a district with a higher unemployment rate or more building approvals. When the number of pension receivers increases, the number of beef cattle owned also increases. The results that more sheep and beef cattle are raised in the areas with lower building approvals or more pension receivers may reflect the fact that pension receivers more often live in the rural areas and new building constructions are lower there.

In Table 4, dairy cattle exhibit a hill-shaped temperature response contrary to the findings for sheep and beef cattle, but only winter temperature terms, linear and quadratic, are significant at 5%. Larger numbers of dairy cattle are raised on Ferralsols, Phaeozems, and Luvisols. The regression reveals a strong socio-economic influence. Fewer dairy cattle are raised in high unemployment zones. An increase in passenger vehicles or building approvals decreases the number of dairy cattle while an increase in pension receivers increases it. An increase in female ratio in the human population decreases the ownership of dairy cattle substantially. Female ratio is higher in major cities. Dairy cattle are only raised in half the towns that raise beef cattle. This is probably due to the higher water requirements involved with raising dairy cattle, e.g., through the Murray Darling basin. This is also confirmed in the positive estimate for the “water body” variable which is only significant in the case of dairy cattle. The Adj Rsq of the model is also low.

Results for the number of pigs are presented in Table 5. Parameter estimates are significant for precipitation and temperature variables. It shows a hill-shaped response with summer temperature. Fewer pigs are owned in Regosols, Vertisols, and Xerosols. The higher the elevation, the more pigs are raised. The smaller the town, the more pigs are raised. Most socio-economic variables are not significant. More building approvals mean fewer pigs are raised. More pension receivers imply more pigs are raised.

A regression on the aggregate livestock sector revenue, which is defined as the total summed revenue from the sales of all livestock species and products, is shown in Table 6. It also includes milk and wool productions. The Adj Rsq is 0.46. The linear terms of winter temperature and summer precipitation are significant at 1%. Livestock revenue is higher in many soils except Xerosols. Revenue is also higher in high elevations or inland locations. The higher the unemployment rate, the lower the livestock revenue. The larger the number of building approvals, the lower the livestock revenue. An area with more pension receivers has higher livestock revenue.

## 5. Impact and Sensitivity Analysis

Marginal impacts of a change in the climate variables are calculated based on the estimated regressions and are shown in Table 7. Based on the full models, if temperature increases by 1 °C, beef cattle and pigs increase with an elasticity of +0.36 and +0.54 respectively. On the other hand, sheep and dairy cattle decrease with an elasticity of −0.16 and −0.34 respectively. On aggregate, livestock revenue increases with an elasticity of +0.27. The results can be contrasted with crop studies which find that in hotter areas some crops are severely harmed by a warmer climate [28,30]. This implies that Australian farmers may increase livestock over crops when temperature becomes hotter, as has been found elsewhere [9,10,17].

When precipitation increases by 1%, beef cattle, sheep, and pigs decline in numbers with an elasticity of −0.07, −0.49, and −0.67 respectively. Dairy cattle increase but only slightly. Thus, wetter conditions decrease livestock populations likely by increasing cropped area as found in [9,10,17]. Furthermore, livestock revenue falls with an elasticity of −0.09. Again, the results can be contrasted with crop studies which report that crops generally benefit from increased rainfall [2,28]. An alternative interpretation is that slightly less rainfall causes more livestock production. Sheep and cattle are also raised more often in drier zones of Africa and Latin America [7,8].

An alternative specification without socio-economic factors leads to similar but slightly different results, as shown in the middle panel of Table 7. When temperature increases, marginal elasticities are slightly smaller with an exception of sheep. When precipitation increases, the elasticity of beef cattle turns positive, an indication of a mis-specification due to omitted household variables. Therefore, the full model should be preferred to the model without social variables.

In the above analyses, the impact of climate change on crops and the availability of feed were not explicitly considered. A hotter and drier climate is beneficial for livestock since it alters the landscape from forests or croplands to pasture suitable for livestock but at the same time, can lead to reduced feed available from grain production. To explicitly test the impact of reduced feed on livestock production, additional regressions were run and are presented in Table 8 by adding a variable for feed availability. The size of the land used for grain production is used as a proxy for feed availability. The regressions for the five dependent variables indicate that feed availability/cropland use does affect the number of each species owned and the aggregate livestock revenue, *i.e.*, the estimates are significant at 5%. Alternatively, livestock management may have increased the demand for crops to be used for feed. Interestingly, feed availability decreases the number of dairy cattle owned while it increases the numbers of other species owned. This implies that dairy cattle are substitutes for grains while beef cattle, sheep, and pigs are complementary goods. That is, if climate conditions are the same, a municipality with a larger grain land raises larger numbers of beef cattle, sheep, and pigs because of feed availability, but not dairy cattle.

Marginal elasticities based on the model including feed availability/cropland use are presented at the bottom panel of Table 7 using the feed availability model in Table 8. There are no significant changes in the elasticities except that precipitation elasticity turned negative in the case of dairy cattle. First, these results imply that the indirect impact of climate change on feed availability is implicitly captured in the full models of Table 2,Table 3,Table 4,Table 5 and Table 6. But the changed sign of dairy cattle elasticity may indicate that dairy cattle and crops often compete for resources such as land and water.

Lastly, Australia is one of the largest livestock exporters in the world with Japan, China, South Korea, Indonesia, and the US being the largest importers of Australian livestock. Over two thirds of Australian agriculture is exported. Agriculture accounts for about 20% of Australian merchandise exports, more than half of which comes from livestock [44]. Given the nature of the cross-sectional data set employed, Australian farmers across different climate zones faced export conditions similar to the survey year 2006. Across the landscape, however, access to export market participation varies a great deal. In our regressions in the previous section, they are captured by the distance to coasts and number of passenger vehicles owned. Estimates in Table 2,Table 3,Table 4,Table 5 and Table 6 indicate that distance to coastal markets and the number of passenger vehicles are not a major factor, *i.e.*, their estimates are not significant. Good road and transportation systems across the country developed for livestock exports, e.g., Australian Livestock Transporters Association (ALTA) and Livestock and Bulk Carriers Association (LBCA), may have eliminated the obstacles of transportation to access ports.

A simple analysis in Table 9 compares the sales of beef cattle, sheep, and wool per farm business in 1990 with those in 2009 using the ABARE surveys since 1990. It is notable that beef cattle sales have increased dramatically over this time period in the Northern Territory and Western Australia, the regions which are climatically unfriendly for humans, due primarily to the improved road and transportation systems. Therefore, improved access to export markets in the future will likely further increase production of livestock in Australia in these remote regions. What will happen to foreign demand is less clear. If climate is shifted globally, overseas demands for Australian livestock may fall due to increased productions in the US, Africa, and Latin America [9,10,17] but foreign demands may increase elsewhere such as Middle East, South Asia, and northern Europe. Decreased international demands will result in increased transportation fees to remote zones since transporters are supposed to charge more, which will lead to decreased productions in these remote grasslands. In our regressions, these effects are partially captured by the coefficients in the distance to coasts.

## 6. Climate Scenarios

The long term impacts of climate change on the livestock sector are examined in this section based on the estimated parameters of the full models. This exercise is meant to single out the sole impacts of climate scenarios on the livestock sector keeping all other factors fixed. Therefore, this exercise excludes the external influences of technological changes, population growth, environmental attitudes, and economic development.

Three General Circulation Model scenarios (GCMs) used in the recent IPCC report [1] were used in this examination. Specifically, the A2 scenarios from the GISS ER model (Goddard Institute for Spatial Studies) [38] and HadGEM1 model of the UKMO (United Kingdom Meteorology Office) [39] were used. These scenarios were obtained from the IPCC's Data Distribution Center. In addition, a high degree warming scenario from the Commonwealth Scientific and Industrial Research Organization (CSIRO) was examined [37]. By 2060, the CSIRO scenario predicts a 3.4 °C increase in annual mean temperature and an 8% reduction in annual precipitation [42]. As shown in Table 10, both the GISS and the UKMO scenarios forecast temperature increases of around 2 °C in both summer and winter seasons for the time period between 2040 and 2070. The UKMO scenario predicts a drier climate in both summer and winter while the GISS scenario predicts an increase in summer rainfall by around 10%.

In Table 11, the impacts of these climate scenarios are calculated for the four livestock species and livestock revenue. The top and middle panels of Table 11 show the impacts of the high degree warming CSIRO scenario using the full models. When temperature increases by as much as 3.4 °C, sheep and beef cattle show substantial increases. Sheep increase by 57%, beef cattle by 240%, and pigs by 9%. The number of sheep increases by a large percentage due to U-shaped temperature response functions (Table 7). On the other hand, dairy cattle fall by 10%. Aggregated over all species, livestock revenue increases by around 62%.

When precipitation decreases by 8%, it is notable that all the species benefit from the drier conditions. Sheep and pig populations increase by around 30%. The increases in beef cattle and dairy cattle are modest. Aggregated across all the species as well as various products such as milk and wool, livestock revenue also increases but merely by 0.06%. The impacts of the changes in both temperature and precipitation result in a population change of +82% for sheep, +242% for beef cattle, −9.5% for dairy cattle, and +40% for pigs, along with a +62% increase in livestock revenue.

In the bottom panel of the Table 11, simulation results from the GISS and UKMO scenarios are presented. Across the four species, the increases are far larger under the UKMO scenario, *i.e.*, a hotter and drier scenario. Sheep increase by 122%, beef cattle by 211%, dairy cattle by 29%, and pigs by 72%. A wetter summer predicted by the GISS scenario decreases the desirability of these species. For example, the expected sheep increase is only 22% while dairy cattle decrease by 2%. Again, dairy cattle may compete against crops under milder and wetter conditions. The overall impact on livestock revenue is not different, however, with around 47% increase in revenue in both cases.

What might be the reasons for the increased numbers and revenue from livestock under hotter and drier conditions? Animals may have adaptive capacity to heat and other environmental stress [21]. Numerous management adjustments may also play a significant role in protecting the animals [22,23]. For example, famers might pursue genetic adaptation such as use of a more heat tolerant Brahman cattle breed [18] or substitute livestock for crops when climate becomes hot and arid [9,10,17].

## 6. Discussion

This paper examines the vulnerabilities of livestock species populations and revenue in Australia to climate change. The analysis was based on 2006 livestock population and sales data of the regional livestock profile in around 1,400 local areas. Four major species were examined: beef cattle, dairy cattle, sheep, and pigs. Livestock products such as wool and milk are also analyzed.

The results show that when temperature increases marginally, beef cattle and pig populations increase. On the other hand, dairy cattle and sheep decrease. Furthermore, across all the species, total livestock revenue per farm increases. When precipitation falls marginally, beef cattle, sheep, and pig populations increase but dairy cattle decrease. Across all species, livestock revenue increases, implying that a drier climate will benefit livestock managers except for those with dairy cattle.

Under projected long term climate change scenarios, the results indicate four species raised would increase by large percentages. Under the hotter and drier UKMO HadGEM1 scenario, sheep would increase by 122%, beef cattle by 211%, dairy cattle by 29%, and pigs by 71%. If, on the other hand, the GISS scenario comes to pass, sheep would increase only by 22% due to a projected wetter summer. In both scenarios, livestock revenue is expected to increase by +47% by around 2060. Under the higher degree warming scenario by the CSIRO, these changes become even larger.

In interpreting the results, several qualifications need to be added. These findings seem to contradict the scientific studies of cattle which indicated decreased weight growth and conception of cattle due to increased heat stress, but are in line with the vegetation studies which predict increased grasslands for livestock [45]. Livestock species may face threshold climate values which are not evident from the regional data used in this study as the temperature could rise beyond the ranges in the data [21]. Secondly, this paper does not determine, due to data limitations, whether alternative breeds of cattle or sheep would be abandoned or favored due to their vulnerability or resilience [18]. Third, goats and chickens were omitted from the analysis as data were not available, so they are conspicuously missing in the analysis. Fourth, whether increased pasture under the hot and arid conditions means low quality of grass and lessened productivity is not fully addressed by the results in this research except to the extent that current climate variations and livestock activities contain such variation. However, the impacts of the changes of a more extreme nature, outside of the range of historic variation, such as 50% drop in rainfall cannot be answered from this research. Fifth, the research does not incorporate the possibility that altered livestock populations may lead to increased greenhouse gas emissions, and increased cost under the carbon tax regime [46]. However, cases exist where methane emissions from livestock can be reduced substantially with little cost by feed management and dietary additives, which may become needed for Australian producers [47]. Finally, there are no available household level micro data that allow examination of household climate adaptation choices including feed purchase alternatives, so this is omitted.

## 7. Conclusion

The findings in this paper show livestock management is likely to be more resilient under a hotter and drier world than are crops. In climate zones like Australia, findings have shown that major crops may be highly vulnerable to climate change [28,30]. The finding that livestock species can be raised successfully under more arid conditions may deserve special attention by farm mangers and policy makers as they weigh adaptation possibilities. This is particularly true in Australia, which has vast areas of grasslands nationwide except for the forested areas along the eastern coasts. This research finds that climate change favors cattle and sheep although this merits local examination in terms of the suitability of species and breeds. The underlying driver of these results involves land use change, lowered disease incidence and intrinsically lower vulnerability relative to crops [45,48-51]. Given that climate change will unfold over the next 100 years, a wise adaptation portfolio which includes the livestock sector is certainly called for.

## Figures and Tables

**Table 1:** Descriptive statistics.

	**NSW (New South Wales)**	**VIC (Victoria)**	**QLD (Queensland)**	**SA (South Australia)**	**WA (Western Australia)**	**TAS (Tasmania)**	**NT (Northern Territory)**	**ACT (Australian Capital Territory)**
Summer temperature (°C)	22.4	19.4	25.4	21.4	24.1	15.1	28.3	18.7
Winter temperature (°C)	10.0	8.7	15.0	10.7	12.2	7.3	22.4	5.4
Summer precipitation (mm/month)	90.2	46.0	146.9	20.7	17.3	62.9	258.7	58.8
Winter precipitation (mm/month)	61.0	74.1	46.3	59.5	84.1	116.6	4.8	65.9
Water body dummy (fraction)	0.1	0.0	0.5	0.0	0.0	0.6	0.4	0.0
Acrisols dummy (fraction)	0.2	0.0	0.0	0.0	0.0	0.0	0.0	0.0
Cambisols dummy (fraction)	0.1	0.1	0.0	0.0	0.0	0.1	0.0	0.0
Ferralsols dummy (fraction)	0.0	0.0	0.0	0.0	0.0	0.0	0.0	0.0
Phaeozems dummy (fraction)	0.0	0.0	0.0	0.0	0.0	0.0	0.0	0.0
Lithosols dummy (fraction)	0.0	0.0	0.0	0.0	0.0	0.0	0.2	0.0
Luvisols dummy (fraction)	0.2	0.3	0.0	0.1	0.1	0.0	0.0	0.0
Nitosols dummy (fraction)	0.1	0.3	0.0	0.0	0.0	0.0	0.0	0.0
Podzols dummy (fraction)	0.0	0.0	0.0	0.0	0.0	0.0	0.0	0.0
Arenosols dummy (fraction)	0.0	0.0	0.0	0.0	0.2	0.0	0.0	0.0
Regosols dummy (fraction)	0.0	0.0	0.0	0.0	0.0	0.0	0.0	0.0
Solonetz dummy (fraction)	0.0	0.1	0.0	0.1	0.1	0.0	0.0	0.0
Vertisols dummy (fraction)	0.1	0.1	0.1	0.0	0.0	0.0	0.0	0.0
Planosols dummy (fraction)	0.1	0.2	0.2	0.0	0.3	0.2	0.4	1.0
Xerosols dummy (fraction)	0.0	0.0	0.0	0.7	0.0	0.0	0.0	0.0
Land size (km^2^)	5,306.5	1,449.4	9,456.8	1,0425.2	1,3446.9	1,736.3	929.9	412.3
Distance coast (km)	182.1	142.6	112.8	119.0	178.5	40.9	187.8	136.3
Elevation (km above sea level)	0.3	0.2	0.2	0.2	0.3	0.3	0.2	0.7
Population (1,000 heads)	2,8221.4	2,0251.4	1,0946.2	9,596.3	9,405.8	1,0475.0	5,023.2	268.8
Female (fraction)	0.49	0.48	0.47	0.48	0.45	0.48	0.40	0.41
Number of passenger vehicles (1,000)	13.38	10.95	4.92	5.27	4.69	5.46	1.97	1.07
Number of bus (1,000)	0.08	0.07	0.04	0.03	0.04	0.04	0.10	0.01
Number of building approvals (1,000)	0.09	0.15	0.08	0.07	0.12	0.05	0.03	0.00
Number of pension receivers (1,000)	2.65	1.76	0.89	1.02	0.66	1.12	0.14	0.01
Total number of observations (N)	149	156	174	84	115	39	5	4

* Acrisols and other soils are a dummy variable for the dominant soil in each local area.

**Table 2:** The number of sheep (in log) per 1,000 rural people.

	**Full Model**		**Without Social Variables**	
	**Mean**	HC* P-Val	**Mean**	**HC P-Val**
Intercept	28.932	<.0001	25.080	<.0001
Summer temperature	−1.243	0.002	−1.070	0.001
Summer temperature sq	0.027	0.003	0.022	0.001
Winter temperature	0.110	0.651	0.044	0.856
Winter temperature sq	−0.007	0.442	−0.0072	0.429
Summer precipitation	−0.077	<.0001	−0.0745	<.0001
Summer precipitation sq	0.0001	<.0001	0.000153	<.0001
Winter precipitation	0.003	0.832	−0.0025	0.850
Winter precipitation sq	−0.0001	0.053	−0.00011	0.095
Water	−0.740	0.134	−0.750	0.162
Acrisols	−1.462	0.015	−2.336	<.0001
Cambisols	−1.765	0.002	−2.0296	<.0001
Ferralsols	0.508	0.365	0.314	0.604
Phaeozems	−0.357	0.687	−0.496	0.551
Lithosols	1.758	0.019	1.681	0.007
Luvisols	−0.355	0.283	−0.345	0.324
Nitosols	−1.266	0.042	−2.588	<.0001
Podozols	0.432	0.283	0.845	0.074
Arenosols	−1.113	0.022	−1.223	0.040
Regosols	0.862	0.133	0.598	0.341
Solonetz	0.501	0.167	0.692	0.059
Vertisols	0.725	0.086	0.853	0.056
Planosols	−0.345	0.356	−0.818	0.053
Xerosols	−1.837	<.0001	−1.618	0.001
Elevation	4.083	<.0001	4.518	<.0001
Land (square km)	0.00001	0.226	0.00326	0.012
Distance to coast (km)	0.004	0.004	−1.5 × 10^−6^	0.781
Unemployment (fraction)	−0.068	0.123		
Female (fraction)	−8.231	0.069		
Number of Passenger vehicles	−0.463	0.030		
Building approvals	−3.335	<.0001		
Pension (fraction)	17.417	<.0001		
Adj Rsq	0.72		0.72	
N	565		565	

* denotes Heteroscedasticity Consistent.

* Units of all the variables are the same as in Table 1.

**Table 3:** The number of beef cattle (in log) per 1,000 rural people.

	**Full Model**		**Without Social Variables**	
	**Mean**	**HC P-Val**	**Mean**	**HC P-Val**
Intercept	15.783	<.0001	16.412	<.0001
Summer temperature	−0.772	0.003	−0.861	0.001
Summer temperature sq	0.017	0.004	0.0181	0.001
Winter temperature	0.211	0.196	0.232	0.206
Winter temperature sq	−0.0003	0.956	−0.00261	0.688
Summer precipitation	−0.002	0.556	0.000456	0.909
Summer precipitation sq	−0.00001	0.289	−1.7 × 10^−5^	0.190
Winter precipitation	−0.008	0.242	−0.0153	0.045
Winter precipitation sq	0.00002	0.425	4.14 × 10^−5^	0.235
Water	−0.772	0.074	−0.939	0.042
Acrisols	−0.764	0.084	−1.463	0.004
Cambisols	−0.615	0.155	−0.915	0.052
Ferralsols	1.168	0.001	1.148	0.006
Phaeozems	0.181	0.632	0.190	0.652
Lithosols	0.108	0.891	−0.348	0.746
Luvisols	0.280	0.308	0.3450	0.253
Nitosols	0.275	0.488	−0.749	0.076
Podozols	1.544	<.0001	1.983	<.0001
Arenosols	−0.925	0.052	−0.685	0.134
Regosols	−0.860	0.008	−1.1547	0.013
Solonetz	0.331	0.364	0.607	0.099
Vertisols	1.121	0.000	1.176	0.000
Planosols	−0.050	0.875	−0.458	0.215
Xerosols	−1.502	<.0001	−1.289	0.001
Land (square km)	0.0002	0.003	1.2 × 10^−5^	0.001
Distance to coasts (km)	0.001	0.610	0.000595	0.632
Elevation	2.548	<.0001	3.0614	<.0001
Unemployment (fraction)	−0.142	<.0001		
Female (fraction)	−3.859	0.319		
Passenger vehicle	−0.011	0.362		
Building approvals	−2.204	<.0001		
Pension (fraction)	17.514	<.0001		
Adj Rsq	0.47		0.39	
N	644		644	

* Units of all the variables are the same as in Table 1.

**Table 4:** The number of dairy cattle (in log) per 1,000 rural people.

	**Full Model**		**Without Social Variables**	
	**Mean**	**HC P-Val**	**Mean**	**HC P-Val**
Intercept	5.702	0.399	−3.862	0.505
Summer temperature	0.698	0.294	0.725	0.273
Summer temperature sq	−0.029	0.080	−0.0289	0.076
Winter temperature	1.106	0.007	1.0037	0.016
Winter temperature sq	−0.035	0.031	−0.0296	0.071
Summer precipitation	0.008	0.325	0.00546	0.491
Summer precipitation sq	0.000	0.379	−1.9 × 10^−5^	0.374
Winter precipitation	−0.021	0.206	−0.0278	0.095
Winter precipitation sq	0.000	0.117	0.000135	0.064
Water	0.385	0.678	0.853	0.364
Acrisols	0.496	0.592	0.143	0.879
Cambisols	1.394	0.140	1.441	0.136
Ferralsols	2.632	0.004	2.914	0.002
Phaeozems	2.305	0.017	2.788	0.004
Lithosols	−1.066	0.489	−1.176	0.455
Luvisols	1.675	0.034	1.931	0.016
Nitosols	0.135	0.889	−0.267	0.785
Podozols	1.416	0.154	2.293	0.022
Arenosols	0.817	0.422	1.006	0.382
Solonetz	0.918	0.439	1.519	0.186
Vertisols	0.925	0.348	1.216	0.238
Planosols	0.412	0.615	0.473	0.567
Xerosols	−0.217	0.800	0.230	0.790
Land (square km)	0.000	0.882	−3.6 × 10^−6^	0.885
Distance to coasts (km)	0.001	0.595	0.000815	0.717
Elevation	−0.087	0.929	0.781	0.445
Unemployment (fraction)	−0.181	0.033		
Female (fraction)	−21.415	0.001		
Passenger vehicle	−0.422	0.003		
Building approvals	−2.787	<.0001		
Pension (fraction)	17.793	0.001		
Adj Rsq	0.19		0.13	
N	364		364	

* Units of all the variables are the same as in Table 1.

**Table 5:** The number of pigs (in log) per 1,000 rural people.

	**Full Model**		**Without Social Variables**	
	**Mean**	**HC P-Val**	**Mean**	**HC P-Val**
Intercept	−4.968	0.419	−5.160	0.325
Summer temperature	1.114	0.031	1.134	0.032
Summer temperature sq	−0.027	0.026	−0.0276	0.027
Winter temperature	0.583	0.097	0.533	0.166
Winter temperature sq	−0.015	0.243	−0.0149	0.286
Summer precipitation	−0.023	0.001	−0.0212	0.001
Summer precipitation sq	0.00002	0.106	2.6 × 10^−5^	0.109
Winter precipitation	−0.084	<.0001	−0.082	<.0001
Winter precipitation sq	0.0003	<.0001	0.00032	<.0001
Water	−1.412	0.051	−1.0912	0.140
Acrisols	−0.456	0.595	−0.872	0.301
Cambisols	−1.667	0.086	−1.606	0.105
Ferralsols	0.587	0.411	0.639	0.394
Phaeozems	−0.796	0.233	−0.361	0.562
Lithosols	−1.199	0.192	−0.731	0.364
Luvisols	−0.456	0.345	−0.193	0.686
Nitosols	0.349	0.665	−0.690	0.420
Podozols	0.301	0.824	1.057	0.367
Arenosols	−0.530	0.399	−0.586	0.329
Regosols	−4.382	<.0001	−4.0458	<.0001
Solonetz	−0.793	0.170	−0.317	0.582
Vertisols	−1.605	0.024	−1.384	0.062
Planosols	0.070	0.891	0.143	0.783
Xerosols	−1.738	0.008	−1.197	0.059
Land (square km)	−0.00004	<.0001	−5.2 × 10^−5^	<.0001
Distance to coasts (km)	0.001	0.695	0.00158	0.399
Elevation	1.847	0.052	2.514	0.008
Unemployment (fraction)	−0.047	0.575		
Female (fraction)	−1.172	0.852		
Passenger vehicle	−0.388	0.873		
Building approvals	−4.562	0.000		
Pension (fraction)	15.271	0.006		
Adj Rsq	0.29		0.25	
N	423		423	

* Units of all the variables are the same as in Table 1.

**Table 6:** Livestock revenue (in log) per 1,000 rural people.

	**Full Model**		**Without Social Variables**	
	**Mean**	**HC P-Val**	**Mean**	**HC P-Val**
Intercept	12.001	<.0001	10.743	<.0001
Summer temperature	−0.297	0.135	−0.3048	0.109
Summer temperature sq	0.005	0.264	0.00503	0.244
Winter temperature	0.332	0.004	0.318	0.012
Winter temperature sq	−0.005	0.253	−0.00503	0.289
Summer precipitation	−0.008	0.005	−0.00747	0.008
Summer precipitation sq	−0.000004	0.605	−4.5 × 10^−6^	0.599
Winter precipitation	−0.006	0.378	−0.0089	0.226
Winter precipitation sq	0.000005	0.876	6.47 × 10^−6^	0.851
Water	−0.275	0.339	−0.343	0.266
Acrisols	0.149	0.635	−0.280	0.391
Cambisols	0.339	0.258	0.113	0.729
Ferralsols	0.812	0.001	0.728	0.011
Phaeozems	0.614	0.050	0.498	0.151
Lithosols	0.750	0.055	0.628	0.105
Luvisols	0.292	0.101	0.275	0.164
Nitosols	0.266	0.393	−0.407	0.201
Podozols	0.661	0.039	0.885	0.018
Arenosols	0.080	0.753	0.107	0.695
Regosols	−0.416	0.316	−0.597	0.241
Solonetz	0.665	0.006	0.765	0.002
Vertisols	0.589	0.007	0.603	0.013
Planosols	0.324	0.144	0.0883	0.721
Xerosols	−0.434	0.072	−0.375	0.145
Land (square km)	+0.000	0.993	8.15 × 10^−7^	0.655
Distance to coasts (km)	0.002	0.000	0.00204	0.0007
Elevation	1.142	0.001	1.579	<.0001
Unemployment (fraction)	−0.088	0.001		
Female (fraction)	−3.491	0.168		
Passenger vehicle	−0.581	0.539		
Building approvals	−1.895	<.0001		
Pension (fraction)	7.643	<.0001		
Adj Rsq	0.46		0.38	
N	559		559	

* Units of all the variables are the same as in Table 1.

**Table 7:** Marginal effects and elasticities.

	**Sheep**	**Beef Cattle**	**Dairy Cattle**	**Pigs**	**Livestock Revenue**
Full Model					
Temperature + 1 °C	−0.16	+0.36	−0.34	+0.54	+0.27
Precipitation + 1%	−0.49	−0.07	+0.02	−0.68	−0.09
Without Social Variables					
Temperature + 1 °C	−0.31	+0.24	−0.28	+0.41	+0.23
Precipitation + 1%	−0.49	+0.09	+0.06	−0.67	−0.11
With Feed Availability					
Temperature + 1 °C	−0.16	+0.37	−0.29	+0.49	+0.27
Precipitation + 1%	−0.44	−0.06	−0.01	−0.62	−0.09

**Table 8:** Analysis of feed availability.

	**Sheep**	**Beef Cattle**	**Dairy Cattle**	**Pigs**	**Livestock revenue**
Intercept	30.299	16.0395	6.717	−4.572	12.301
Summer temperature	−1.384	−0.806	0.526	1.105	−0.325
Summer temperature sq	0.0301	0.0175	−0.0238	−0.0272	0.00568
Winter temperature	0.0564	0.209	1.185	0.508	0.3306
Winter temperature sq	−0.00385	−5.4 × 10^−5^	-0.0394	−0.01204	−0.00504
Summer precipitation	−0.0739	−0.00176	0.00547	−0.0206	−0.00752
Summer precipitation sq	0.000143	−1 × 10^−5^	−9.8 × 10^−6^	2.17 × 10^−5^	−6.1 × 10^−6^
Winter precipitation	0.00918	−0.00687	−0.0220	−0.0785	−0.0056
Winter precipitation sq	−0.00013	0.000022	0.000114	0.000328	4.91 × 10^−6^
Water	−0.703	−0.751	0.464	−1.3901	−0.285
Acrisols	−1.253	−0.721	0.5022	−0.337	0.169
Cambisols	−1.706	−0.594	1.439	−1.599	0.34006
Ferralsols	0.7504	1.222	2.616	0.7207	0.84301
Phaeozems	−0.1305	0.235	2.313	−0.752	0.626
Lithosols	1.8807	0.147	−1.0734	−1.121	0.752
Luvisols	−0.296	0.297	1.729	−0.418	0.295
Nitosols	−1.139	0.298	0.196	0.386	0.31005
Podozols	0.522	1.5705	1.5105	0.386	0.657
Arenosols	−1.217	−0.941	0.91406	−0.57809	0.0624
Regosols	0.508	−0.914		−4.607	−0.4941
Solonetz	0.521	0.341	0.955	−0.744	0.658
Vertisols	0.714	1.125	0.948	−1.556	0.584
Planosols	−0.201	−0.0127	0.436	0.173	0.328
Xerosols	−1.7608	−1.484	−0.109	−1.803	−0.445
Elevation	4.0774	2.28 × 10^−5^	6.12 × 10^−6^	−4.7 × 10^−5^	4.96 × 10^−7^
Land (square km)	−1.1 × 10^−5^	0.000499	0.00142	−2.3 × 10^−5^	0.00194
Distance to coasts (km)	0.00354	2.572	−0.07901	1.819	1.103
Unemployment (%)	−0.04859	−0.1395	−0.177	−0.0341	−0.084
Female (%)	−8.989	−3.9803	−20.883	−2.0263	−3.501
Passenger vehicle	−0.476	−0.0109	−0.425	−0.514	−0.619
Building approvals	−3.0724	−2.10218	−2.838	−4.265	−2.0407
Pension (%)	17.215	17.453	17.9306	15.532	7.553
Feed Availability (size of grain land, 1,000 km^2^)	0.00000666	0.00000131	−0.0000038	0.00000494	0.00000103
Adj Rsq	0.77	0.47	0.19	0.30	0.47
N	561	640	364	423	555

* Units of all the variables are the same as in Table 1.

* Significance of the estimated parameters (P-values) remained similar to those of the full models in Table 2,Table 3,Table 4,Table 5 and Table 6.

**Table 9:** Livestock sales per farm business in Australian regions in 1990 and 2009.

**State and Territory**	**Region**	**Beef cattle sold (no.)**	**Sheep sold (no.)**	**Total wool sold (kg)**	**Beef cattle sold (no.)**	**Sheep sold (no.)**	**Total wool sold (kg)**
		Year = 1990			Year = 2009		
Australia	Average	239	530	11,892	582	503	5,021
	Australia Total	7,178	15,926	356,779	19,222	16,599	165,702
NSW	Central West	38	752	10,465	72	742	6,190
	Coastal	164	17	107	146	36	257
	Far West	85	2,286	47,176	46	1,682	15,357
	North West Slopes and Plain	144	778	9512	117	395	3826
	Riverina	47	689	13,154	112	831	6,483
	Tablelands (Northern Central)	108	797	10,134	115	592	6,209
NT	Alice Springs Districts	1,338	10	35	1,468	0	0
	Barkly Tablelands	0	0	0	7,443	0	0
	Top End Darwin and the Gulf	0	0	0	191	0	0
	Victoria River District	0	0	0	2,034	0	0
QLD	Cape York and the Queensland	478	0	0	655	0	0
	Central North	295	47	9,134	613	13	1,356
	Charleville - Longreach	296	604	19,877	428	593	8,326
	Darling Downs and Central H	192	205	5,326	288	69	649
	Eastern Darling Downs	107	129	1,824	80	89	826
	North Queensland Coastal	278	0	0	161	1	10
	South Queensland Coastal	139	0	0	176	0	0
	West and South West	679	1104	4,0874	672	434	12,636
SA	Eyre Peninsula	2	209	7,979	2	509	6,156
	Murray Lands and Yorke Peninsula	6	484	7,542	15	468	4,822
	North Pastoral	252	744	3,3558	106	1,459	2,0581
	South East	76	937	12,746	133	941	7,261
TAS	Tasmania	122	678	11,515	112	670	5,676
VIC	Central North	34	484	6,810	34	490	3,503
	Mallee	15	716	4,931	14	458	1,350
	Southern and Eastern Victor	68	520	7,357	91	654	5,550
	Wimmera	11	494	7,198	12	595	5,423
WA	Central and South Wheat Belt	29	857	20,754	87	1,318	13,444
	North and East Wheat Belt	5	909	16,608	26	810	7,714
	Pilbara and the Central Past	55	310	30,524	296	1,357	10,180
	South West Coastal	141	583	11,613	113	803	6,516
	The Kimberly	1,881	0	0	3,238	0	0

* A separate regional definition for ACT was not available in the ABARE data set.

* Total number of surveyed farms is around 1,800 for each year.

**Table 10:** General Circulation Models (AOGCMs).

	**UKMO HadGEM1**	**GISS ER**	**CSIRO High Warming**
Summer temperature (°C)	+2.15	+1.91	+3.4
Winter temperature (°C)	+1.86	+2.09	
Summer precipitation (mm/month)	−1.17	7.44	−8%
Winter precipitation (mm/month)	−4.60	−2.80	

**Table 11:** The impacts of climate scenarios by 2060.

	**Sheep**	**Beef Cattle**	**Dairy Cattle**	**Pigs**	**Livestock Revenue (1,000AUD)**
Results under CSIRO					
Species population/Revenue (per 1,000 rural people)	106,973 head	16,644 head	395 head	496 head	$13,460
Temperature + 3.4 °C	+56.7%	+240%	−10.8%	+8.6%	+61.8%
Rainfall −8%	+25.1%	+2%	+1.3%	+31.6%	+0.06%
Temperature +3.4 °C & Rainfall −8%	+81.8%	+242%	−9.5%	+40.2%	+61.9%
GCM Scenarios					
GISS	+20.7%	+162.4%	−2.4%	+54.7%	+47.3%
UKMO	+122.6%	+211.3%	+29.1%	+71.5%	+47.1%

* % denotes percentage changes from the baselines.
